# Endoscopic submucosal dissection of early gastric neoplasms in the fornix using the newly developed scissor‐type SB knife GX

**DOI:** 10.1111/den.12979

**Published:** 2017-11-27

**Authors:** Yuki Sumida, Toshio Kuwai, Sauid Ishaq

**Affiliations:** ^1^ Department of Gastroenterology National Hospital Organization Kure Medical Center and Chugoku Cancer Center Kure Japan; ^2^ Gastroenterology Department Dudley Group Hospitals Birmingham City University Birmingham UK; ^3^ St George's University Grenada West Indies

## Abstract

Watch a video of this article

## Brief Explanation

Endoscopic submucosal dissection (ESD) using a scissor‐type knife, the Stag‐Beetle knife (SB knife; Sumitomo Bakelite, Tokyo, Japan), is considered technically easy and safe as a result of its ability to grasp the target tissue to allow controlled resection without the complex endoscopic tip control required with conventional ESD devices. There are two types of SB knife (short‐type and Jr‐type) used mainly for esophageal and colorectal neoplasms.^1^, ^2^, ^3^ Recently, a new type, SB knife GX, has been developed for gastric ESD. The ridged base of the blades helps grasp tissue more tightly. More details about the characteristics of SB knife GX and comparison with another scissor‐type knife, Clutch Cutter (Fujifilm, Tokyo, Japan),^4^ are described in Video S1.

Gastric ESD in the fornix is considered challenging because it is difficult to maintain the appropriate scope tip position, and the risk of perforation is higher because of the thin wall.^5^ We present this case of ESD for an early gastric neoplasm in the fornix using the SB knife GX.

A 68‐year‐old man was referred for ESD of a slightly elevated neoplasm (0‐IIa) measuring approximately 10 mm in the fornix of the stomach. After injection of sodium hyaluronate into the submucosa, a mucosal incision was made with the SB knife GX. After circumferential incision, we continued submucosal dissection with the same knife. As the scope tip approached closer to the lesion, it was difficult to achieve an accurate position, but rotation of the knife and its ability to grasp tissue before cutting allowed safe ESD without requiring complex manipulation of the scope tip. In addition, the same knife was used for hemostasis. Histopathological examination showed well‐differentiated tubular adenocarcinoma, and part of the tumor infiltrated the submucosa (SM1/195 μm). This was regarded as curative resection. The patient was discharged later without adverse events.

Authors declare no conflicts of interest for this article.

## Supporting information


**Video S1** Characteristics of the SB knife GX (Sumitomo Bakelite, Tokyo, Japan) and the endoscopic submucosal dissection (ESD) procedure using the knife for a 10‐mm 0‐IIa lesion in the fornix (fundus) of the stomach.

## References

[1] Kuwai T , Yamaguchi T , Imagawa H *et al*. Endoscopic submucosal dissection of early colorectal neoplasms with a monopolar scissor‐type knife: Short‐ to long‐term outcomes. Endoscopy 2017; 49: 913–8. 28743145 10.1055/s-0043-113631

[2] Fujinami H , Hosokawa A , Ogawa K *et al*. Endoscopic submucosal dissection for superficial esophageal neoplasms using the stag beetle knife. Dis. Esophagus 2014; 27: 50–4. 23442212 10.1111/dote.12039

[3] Homma K , Otaki Y , Sugawara M , Kobayashi M . Efficacy of novel SB knife Jr examined in a multicenter study on colorectal endoscopic submucosal dissection. Dig. Endosc. 2012; 24(Suppl 1): 117–20. 10.1111/j.1443-1661.2012.01266.x 22533765

[4] Akahoshi K , Motomura Y , Kubokawa M *et al*. Endoscopic Submucosal Dissection for Early Gastric Cancer using the Clutch Cutter: a large single‐center experience. Endosc. Int. Open 2015; 3: E432–8. 26528497 10.1055/s-0034-1392509 PMC4612246

[5] Minami S , Gotoda T , Ono H , Oda I , Hamanaka H . Complete endoscopic closure of gastric perforation induced by endoscopic resection of early gastric cancer using endoclips can prevent surgery (with video). Gastrointest. Endosc. 2006; 63: 596–601. 16564858 10.1016/j.gie.2005.07.029

